# Association Between Socioeconomic Status and Adherence to Fecal Occult Blood Tests in Colorectal Cancer Screening Programs: Systematic Review and Meta-Analysis of Observational Studies

**DOI:** 10.2196/48150

**Published:** 2023-10-31

**Authors:** Zilin Luo, Xuesi Dong, Chenran Wang, Wei Cao, Yadi Zheng, Zheng Wu, Yongjie Xu, Liang Zhao, Fei Wang, Jibin Li, Jiansong Ren, Jufang Shi, Wanqing Chen, Ni Li

**Affiliations:** 1 Office of Cancer Screening National Cancer Center/National Clinical Research Center for Cancer/Cancer Hospital, Chinese Academy of Medical Sciences and Peking Union Medical College Beijing China; 2 Chinese Academy of Medical Sciences Key Laboratory for National Cancer Big Data Analysis and Implement Chinese Academy of Medical Sciences and Peking Union Medical College Beijing China; 3 Department of Epidemiology and Biostatistics, Jiangsu Key Lab of Cancer Biomarkers, Prevention and Treatment, Collaborative Innovation Center for Cancer Personalized Medicine School of Public Health Nanjing Medical University Nanjing China

**Keywords:** adherence, colorectal cancer, fecal occult blood test, screening, socioeconomic status

## Abstract

**Background:**

Screening adherence is important in reducing colorectal cancer (CRC) incidence and mortality. Disparity in CRC screening adherence was observed in populations of different socioeconomic status (SES), but the direction and strength of the association remained unclear.

**Objective:**

We aimed to systematically review all the observational studies that have analyzed the association between SES and adherence to organized CRC screening based on fecal occult blood tests.

**Methods:**

We systematically reviewed the studies in PubMed, Embase, and Web of Science and reference lists of relevant reviews from the inception of the database up until June 7, 2023. Individual SES, neighborhood SES, and small-area SES were included, while any SES aggregated by geographic areas larger than neighbors were excluded. Studies assessing SES with any index or score combining indicators of income, education, deprivation, poverty, occupation, employment, marital status, cohabitation, and others were included. A random effect model meta-analysis was carried out for pooled odds ratios (ORs) and relative risks for adherence related to SES.

**Results:**

Overall, 10 studies, with a total of 3,542,379 participants and an overall adherence rate of 64.9%, were included. Compared with low SES, high SES was associated with higher adherence (unadjusted OR 1.73, 95% CI 1.42-2.10; adjusted OR 1.53, 95% CI 1.28-1.82). In the subgroup of nonindividual-level SES, the adjusted association was significant (OR 1.57, 95% CI 1.26-1.95). However, the adjusted association was insignificant in the subgroup of individual-level SES (OR 1.46, 95% CI 0.98-2.17). As for subgroups of the year of print, not only was the unadjusted association significantly stronger in the subgroup of early studies (OR 1.97, 95% CI 1.59-2.44) than in the subgroup of late studies (OR 1.43, 95% CI 1.31-1.56), but also the adjusted one was significantly stronger in the early group (OR 1.86, 95% CI 1.43-2.42) than in the late group (OR 1.26, 95% CI 1.14-1.39), which was consistent and robust. Despite being statistically insignificant, the strength of the association seemed lower in studies that did not adjust for race and ethnicity (OR 1.31, 95% CI 1.21-1.43) than the overall estimate (OR 1.53, 95% CI 1.28-1.82).

**Conclusions:**

The higher-SES population had higher adherence to fecal occult blood test–based organized CRC screening. Neighborhood SES, or small-area SES, was more competent than individual SES to be used to assess the association between SES and adherence. The disparity in adherence between the high SES and the low SES narrowed along with the development of interventions and the improvement of organized programs. Race and ethnicity were probably important confounding factors for the association.

## Introduction

Worldwide, colorectal cancer (CRC) ranks third in cancer incidence and second in cancer mortality [1]. It has been confirmed that screening with fecal occult blood tests (FOBT) is effective in lowering CRC mortality in randomized controlled trials [2-4] and in the real world [5-7]. Unlike opportunistic screening programs, organized projects actively invite all individuals within a given age range in a specific geographic area to participate in protocol-based screening programs [8,9]. International academic organizations highly recommend the implementation of organized screening programs to reduce the burden of CRC [10-13].

The benefits of screening depend highly on screening compliance [14]. What is more, extensive research showed that the lower–socioeconomic status (SES) population had a higher incidence of CRC and even higher mortality due to poorer treatment [15-18], which meant there was a greater need for high screening adherence in the low SES to reduce health inequity. Although previous evidence suggested that inequities of income observed in opportunistic programs could largely be eliminated in organized programs [19], the association between SES and organized CRC screening adherence was inconsistent across studies [9].

A review conducted by de Klerk et al [9] qualitatively assessed the association of SES and individuals’ adherence to FOBT-based organized CRC screening programs. This review found that among 11 programs, 90% (28/31) of publications reported lower adherence in the lower socioeconomic population. However, there were distinct differences in how SES was measured. Most used some types of indexes of deprivation, while some studies used more than one indicator to assess SES [9]. Currently, there were no data on the direction and strength of the association between SES and FOBT-based organized CRC screening adherence. In addition, although differences in adherence by SES and race and ethnicity had received much attention and racial disparities were partially explained by differences in SES [20], the role of race in the association of SES with adherence was controversial [21-23]. What is more, with socioeconomic disparity coming under the spotlight, efforts should be made to narrow it, and its changing trend deserves continued attention [9].

There is no conclusive evidence on the association between SES and adherence in FOBT-based organized screening. It is warranted to examine the association between SES and adherence, contributing to the improvement of screening adherence and equity to promote health equity in screening programs. Thus, the study aimed to perform a meta-analysis focusing on the association between SES and FOBT-based organized CRC screening adherence.

## Methods

### Search Strategy and Selection

We conducted the systematic review and meta-analysis under the direction of the PRISMA (Preferred Reporting Items for Systematic Reviews and Meta-Analyses) statement [24]. We searched PubMed, Embase, and Web of Science from the inception date to June 7, 2023, using the search strategy: (socioeconomic OR “socioeconomic status” OR SES OR income OR education OR occupation OR insurance OR “social status”) AND (colorecta* OR bowel) AND cancer* AND (screen* OR “early diagnosis” OR “early detection”) AND (organized OR program*) AND (uptake OR adherence OR nonadherence OR compliance OR noncompliance OR participation OR participating OR nonparticipation OR attendance OR nonattendance OR engagement OR determine* OR factor* OR associate*). The integrated search strategy is shown in Table S1 in Multimedia Appendix 1, and references cited in review articles that were on similar topics were also checked.

### Inclusion and Exclusion Criteria

First, titles and abstracts, and then full-text articles, were screened for inclusion by 2 investigators (ZL and CW) independently. We included studies that measure the association between SES and adherence to organized CRC screening programs. Inclusion and exclusion criteria are shown in Textbox 1.

**Inclusion and exclusion criteria of studies.**

**Inclusion criteria**

- Original articles
- Observational studies conducted in organized colorectal cancer (CRC) screening programs
- Used fecal occult blood tests (FOBT) as the only primary screening test
- Average-risk population
- Assessed socioeconomic status (SES), including individual SES, neighborhood SES, and small-area SES
- Assessed SES with any index or score combining indicators of income, education, deprivation, poverty, occupation, employment, marital status, cohabitation, and others, and the SES index or score was defined as categorical variables
- Provided data to obtain related either unadjusted effect sizes (including odds ratios and relative risks) or adjusted ones
- Assessed adherence or nonadherence to FOBT-based CRC screening, which was objectively recorded
- Assessed adherence rate with the percentage of individuals invited to participate in screening that participate
- English language

**Exclusion criteria**

- Lacked enough data to calculate either unadjusted or adjusted effect sizes, including qualitative studies, reviews, commentaries, letters, and guidelines
- Conference abstracts that did not contain enough information for quality assessment
- Assessed adherence to colonoscopy or other screening tests
- Focused on only low-SES population or high-SES population, for example, focusing on only low-income population
- If several studies were conducted in the same program, participants should be counted only once. Duplicated samples were excluded, and the study with the biggest sample that offered the most information was included.
- Assessed country-level SES, province-level SES, or any SES aggregated by geographic areas larger than neighbors
- Assessed self-reported adherence
- Any language other than English

### Data Extraction and Statistical Analysis

Sample data were extracted by 2 investigators (ZL and CW) independently, with disagreements solved by discussion and consensus. The standardized form consists of (1) the first author; (2) publication year; (3) study design; (4) country; (5) program name; (6) sample size; (7) adherence rate; (8) name of the SES index or score; (9) SES measures involved; (10) focusing on SES or not; (11) SES level; (12) original adjusted effect size; (13) available unadjusted and adjusted effect sizes (relative risks were transformed into odds ratios [ORs]) [25]; (14) covariates of adjusted effect sizes; (15) single-level or multilevel multivariate model; and (16) age range of screening. If effect sizes of an SES index or score with more than 2 levels were reported (usually), the effect sizes between the 2 extremes were extracted (eg, most vs least deprivation). The lowest SES was used as the reference group, and if the highest SES was used as the reference group, the reference group of the OR would be converted. When the unadjusted effect sizes were absent, they were calculated with the present cross-table data.

The Newcastle-Ottawa Quality Assessment Scale (NOS) for cohort studies and the Agency for Healthcare Research and Quality (AHRQ) methodology checklist were separately used to assess the quality of cohort and cross-sectional studies, which categorized the studies as high, middle, and low-quality [26-28]. The pair of investigators rated the quality independently, and discrepancies were solved through discussion and consensus. The Kappa coefficient was used to evaluate the consistency of the results.

### Data Synthesis

ORs with corresponding 95% CIs were used for analysis, and an inverse variance method was used. We used Stata (version 17.0; StataCorp) to analyze data. The “flocci” command was used to convert the reference group of original estimates. “mean,” “meta bias,” and “meaning” were used to perform analysis. The Cochrane *Q* statistics and *I*^2^ estimations were used to assess study heterogeneity. With considerable heterogeneity, a random effect model using the Daimonian and Laird method was used to conduct the meta-analysis.

We first assessed the unadjusted bivariate correlations between SES and adherence. Then we used the effect sizes of multivariate models with the most adjustment factors to assess the adjusted association. Tables and forest plots were used to display the results.

We examined heterogeneity using the Cochrane *Q* statistic and the *I*^2^ statistic. Subgroup analysis was conducted to probe the potential source of heterogeneity across studies according to some characteristics of the studies: year of print (before or after the median year of print of the studies), adherence rate (lower or higher than 45%, which was the acceptable threshold set by the European guidelines [14]), SES focus (whether focusing on SES or other factors), SES level (individual level or nonindividual level, which includes neighborhood and small-area SES), and adjustment (adjusting for race and ethnicity or not, only for adjusted effect sizes) with at least 3 studies in a group. Publication bias was assessed using Egger and Begg tests [29,30]. Sensitivity analysis investigated the influence of each study (including the only cross-sectional study) on the overall meta-analysis summary estimate through a random effect model using the method of Daimonian and Laird.

## Results

### Systematic Review

**Figure 1:**
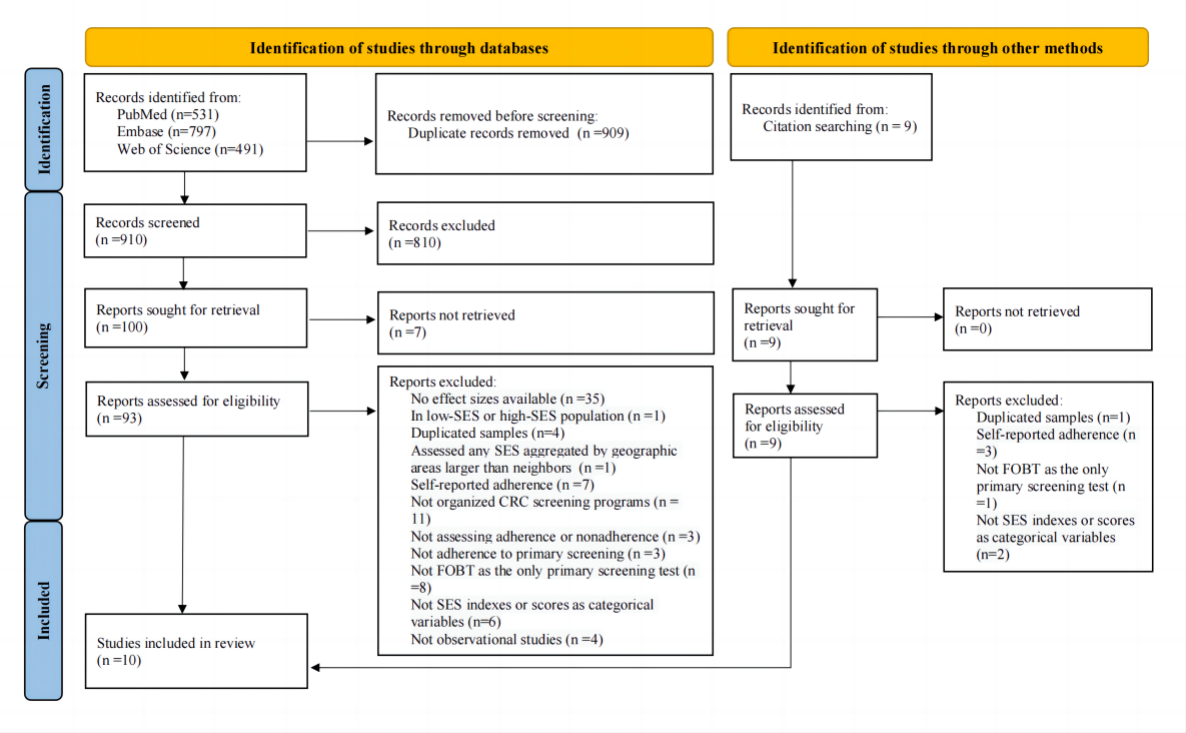
PRISMA (Preferred Reporting Items for Systematic Reviews and Meta-Analyses) diagram. CRC: colorectal cancer; FOBT: fecal occult blood test; SES: socioeconomic status.

The initial search yielded 1819 articles, and 9 articles were identified from reference lists. Of the 1819 articles, 910 remained after removing 909 duplicates by EndNote X8.1 (Thompson Reuters) automatically (Figure 1) [24,31]. In total, 102 full-text articles were assessed, and 10 met the inclusion and exclusion criteria.

Table 1 shows an overview of the included studies, and Table S2 in Multimedia Appendix 1 lists other characteristics. A total of 9 of them were cohort studies, while 1 (515,388 participants) was cross-sectional. All studies included were carried out in developed European countries. All of the studies were of high quality, and the consistency of the literature quality evaluation results was 1.00 (Tables S3 and S4 in Multimedia Appendix 1). There was no uniform adjustment, and the potential confounders, including age, sex, race, ethnicity, cohabitation, marriage, and employment, were included and adjusted in different ways. Surprisingly, all ORs included were significantly larger than 1.00; in other words, all the studies agreed that SES was positively associated with adherence. Therefore, the results of the comparison of unadjusted and adjusted pooled estimates between subgroups were more important.

**Table 1:** Overview of the studies included in the meta-analysis.

Reference	Country	Program	SES^a^ index or score	SES measures involved	SES focus^b^	SES level	Multivariate model
Poncet et al (2013) [32]	France	—^c^	Townsend Index	Employment, car ownership, home ownership, and overcrowded housing units	No	Nonindividual	Multilevel
Pornet et al (2010) [33]	France	—	Townsend Index	Employment, car ownership, home ownership, and overcrowded housing units	Yes	Nonindividual	Multilevel
Solís-Ibinagagoitia et al (2020) [34]	Spain	Bowel Cancer Screening Programme of the Basque Country	Deprivation Index	Education, occupation, and employment	No	Individual	Single-level
Steele et al (2010) [35]	United Kingdom	—	Scottish Index of Multiple Deprivation	Education, income, employment, housing, health, and access to facilities	Yes	Nonindividual	—
Szczepura et al (2008) [36]	United Kingdom	English Bowel Cancer Screening Pilot	The Carstairs Index of Deprivation	Employment, car ownership, overcrowding, and social class	No	Individual	Single-level
van der Meulen et al (2022) [37]	Netherlands	Dutch national CRC^d^ screening programme	SES scores based on income, education, and employment	Education, income, and employment	Yes	Nonindividual	Single-level
van der Vlugt et al (2017) [38]	Netherlands	—	SES scores based on income, education, and employment	Income, education, employment, and position on the labor market	No	Nonindividual	—
Ward et al (2011) [39]	Australia	National Bowel Cancer Screening Program	Index of Relative Social Disadvantage	Education, income, and employment	No	Nonindividual	Single-level
Weller et al (2007) [40]	United Kingdom	UK Colorectal Cancer Screening Pilot	Index of Multiple Deprivation	Education, income, employment, health and disability, barriers to housing and services, crime, and living environment	No	Nonindividual	Single-level
Buron et al (2017) [41]	Spain	Barcelona colorectal cancer screening programme	Medea Deprivation Index	Education, occupation, and employment	Yes	Individual	Single-level

^a^SES: socioeconomic status.

^b^SES focus: whether focusing on socioeconomic status or other factors.

^c^Not available.

^d^CRC: colorectal cancer.

### Meta-Analysis With Unadjusted and Adjusted Effect Sizes

A total of 9 studies provided unadjusted effect sizes, with 1,927,075 (63.7%) participants in 3,026,991 targeted individuals. And 8 studies reported adjusted effect sizes, involving 3,220,822 targeted individuals and 2,117,839 (65.8%) participants.

**Figure 2:**
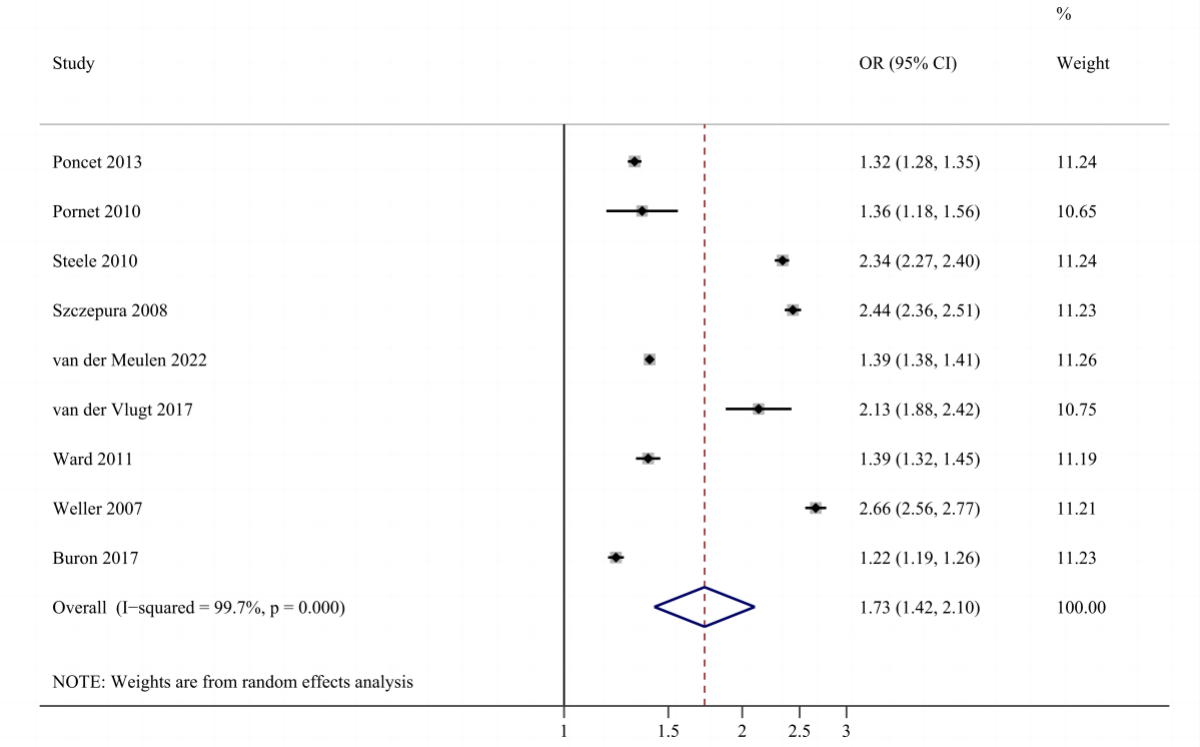
Forest plot of total unadjusted effect sizes for the association between socioeconomic status and adherence. OR: odds ratio.

A positive and strong association was observed between SES and adherence (unadjusted OR 1.73, 95% CI 1.42-2.10; adjusted OR 1.53, 95% CI 1.28-1.82). The forest plots are shown in Figures 2 [32,33,35-41] and 3 [32-34,36,37,39-41]. There was no evidence of publication bias. Sensitivity analysis showed robust results (Figures S1 and S2 in Multimedia Appendix 1). A fair degree of heterogeneity was found.

**Figure 3:**
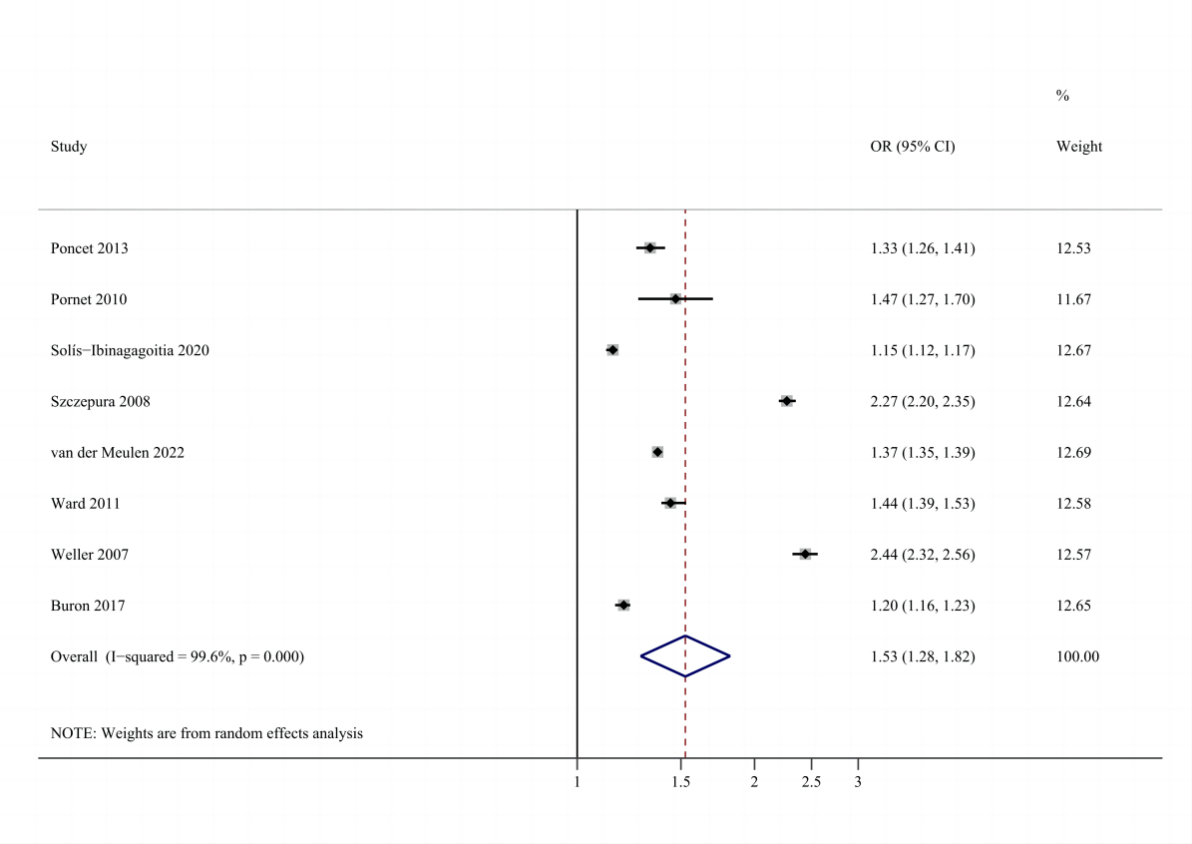
Forest plot of total adjusted effect sizes for the association between socioeconomic status and adherence. OR: odds ratio.

### Subgroup Analyses With Unadjusted and Adjusted Effect Sizes

**Figure 4:**
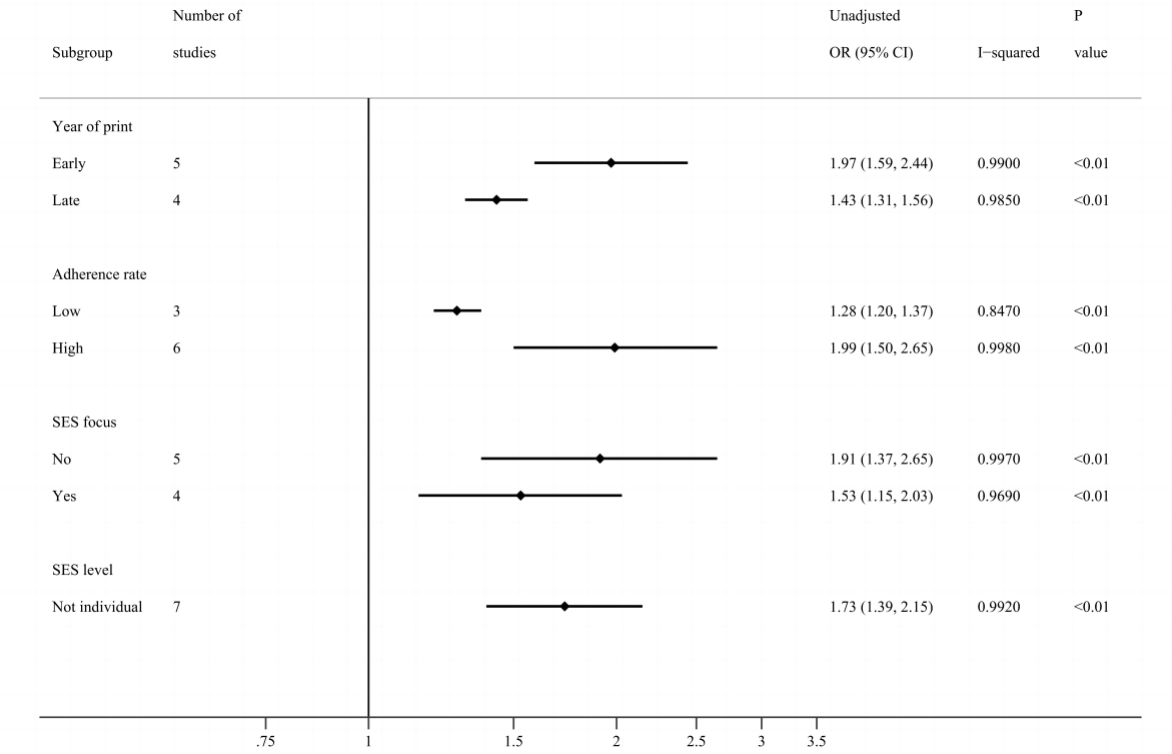
Forest plot of subgroup analysis using unadjusted effect sizes. There were only 2 studies in the subgroup of individual-level socioeconomic status (SES), which were not included in the subgroup analysis. *P* values less than .05 were considered statistically significant. OR: odds ratio; SES focus: whether focusing on socioeconomic status or other factors.

**Figure 5:**
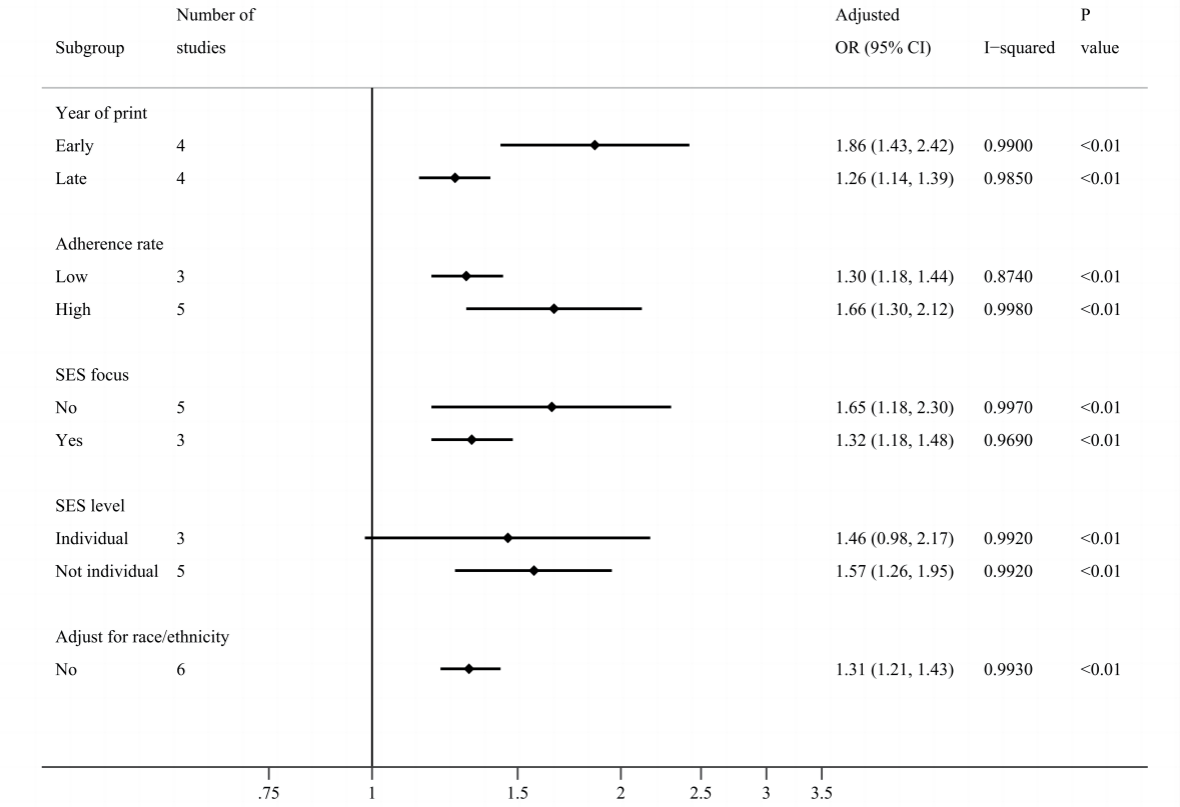
Forest plot of subgroup analysis using adjusted effect sizes. There were only 2 studies that adjusted for race and ethnicity, which were not included in the subgroup analysis. OR: odds ratio; SES focus: whether focusing on socioeconomic status or other factors.

The positive association between SES and adherence remained significant in all feasible subgroup analyses (with at least three studies in a group), except for the adjusted association in the subgroup of individual-level SES (adjusted OR 1.46, 95% CI 0.98-2.17; Figures 4 and 5; Figure S3 in Multimedia Appendix 1). On the contrary, in subgroups of nonindividual-level SES, the adjusted association was significant. In other words, high nonindividual SES increased adherence, but individual SES did not influence adherence.

As for subgroups of the year of print, not only was the unadjusted association significantly stronger in the subgroup of early studies (OR 1.97, 95% CI 1.59-2.44) than in late studies (OR 1.43, 95% CI 1.31-1.56; Figure S4 in Multimedia Appendix 1), but also the adjusted one was significantly stronger in the early group (OR 1.86, 95% CI 1.43-2.42) than the late group (OR 1.26, 95% CI 1.14-1.39; Figure S5 in Multimedia Appendix 1), which was consistent and robust. In addition, the overall adherence rate of the late studies was higher than the early ones, which were 67.2% versus 52.1% in unadjusted associations and 68.1% versus 50.1% in adjusted associations.

The other results were not statistically significant, but they provided some insights. For multivariate analysis, the pooled estimate of the subgroup of studies that did not adjust for race and ethnicity (OR 1.31, 95% CI 1.21-1.43) seemed smaller than the overall pooled estimate (OR 1.53, 95% CI 1.28-1.82). The adjustment for race and ethnicity better showed a positive association. In addition, the strength of the association between SES and adherence seemed lower when the adherence rate was low or when SES was the focus of the studies (unadjusted OR 1.53, 95% CI 1.15-2.03; adjusted OR 1.32, 95% CI 1.18-1.48) than not (unadjusted OR 1.91, 95% CI 1.37-2.65; adjusted OR 1.65, 95% CI 1.18-1.48).

## Discussion

### Meta-Analytic Findings

To our knowledge, this is the first systematic review and meta-analysis assessing the strength of the association between SES and FOBT-based organized CRC screening adherence. It indicated considerably higher adherence among the highest SES compared with the lowest. The positive association was significant in both unadjusted and adjusted analyses, while being consistent across most possible sets of pooled estimates and all possible subgroup analyses, including the year of print, adherence rate, SES focus, SES level, and adjustment for race and ethnicity.

The association between high SES and high adherence was similar to those reported in 4 previous reviews investigating the participation of multiple modalities of CRC screening in both organized and opportunistic screening programs [42-45]. Furthermore, the disparity in adherence would lead to disparity in CRC incidence and mortality. In a modeling study estimating the disparity between African American and White individuals in the United States, the disparities in uptake of screening were able to account for 42% of the disparity in incidence and 19% of the disparity in mortality [46]. It thus seemed urgent to decrease the disparity in adherence to reduce health inequity driven by SES.

### Differences in Subgroup Analysis

Subgroup analysis of different SES levels indicated that individual-level SES was not associated with adherence, which suggested that different SES levels could be the source of heterogeneity between studies. A systematic review by Mosquera et al [47] indicated that different indicators of individual SES showed various associations with adherence, separately. While individual educational level was positively associated with screening participation and higher deprivation was associated with lower participation, studies differed in the results of the association between individual income and participation [47]. In this meta-analysis that assessed SES index and scores rather than single indicators, individual SES was not associated with adherence after adjustment.

Nevertheless, country-level SES, which was not involved in this meta-analysis, was confirmed to not be associated with adherence either. In 2009, Pruitt et al [48] conducted a systematic review of the association between area SES and adherence. They found that 3 of 5 studies had at least one positive association and noted that country-level SES variables were not associated with any type of CRC screening adherence. Therefore, adherence might be associated with neighborhood or small area–level SES rather than individual- or country-level [48].

Due to the difficulty and trouble of collecting individual-level data, commonly used measures of SES were based on geography, at different levels of aggregation from countries to neighborhoods [49]. And it was proven that the agreement between area- and individual-level SES was low [50]. As a result, neighborhood SES or small-area SES was more competent to be used to assess the association between SES and adherence, and in this case, SES was significantly associated with adherence.

Although socioeconomic and racial and ethnic disparities in organized CRC screening were noted [9,51], the effect of race and ethnicity on the association has not been concluded yet. Another important finding of this analysis was that race and ethnicity might be important confounding factors for the association between SES and adherence. In accordance with the present results, a previous study in the United States using income level as the measure of SES reported that in the hierarchical analysis by race and ethnicity, there was no significant association between SES and adherence among the non-White population. Especially for adherence to a screening colonoscopy, a significant interaction between race and ethnicity and income level was identified [52]. Although O’Malley et al [21] declared that racial differences in adherence could be fully explained by differences in SES, most studies of other conditions found independent associations with both SES and race and ethnicity [22,23]. Consequently, in order to improve adherence and address inequity, future research should consider strategies to improve the adherence of the low-SES population regarding ethnic composition.

The results of this meta-analysis showed that the disparity between the high and low-SES populations in adherence decreased. Since the adherence rate of the subgroup with a later year of print was higher than that of the other subgroup, a probable explanation was that adherence of the low-SES population increased more than the high as organizational screening programs developed with more and more interventions to promote adherence. Actually, a large-sample randomized controlled trial proved that certain interventions could not only improve overall participation but also particularly enhance the adherence of the low-SES population [53]. In this context, future studies should focus on cost-effective strategies that are suitable to be integrated into large-scale organized programs and further address the disparity in the near future. Our ideal goal is not equal adherence but to eliminate the health inequity of the CRC.

### Strengths and Limitations

This is the first meta-analysis assessing the association between SES and adherence, especially with the first focus on FOBT-based organized CRC screening adherence. Previous reviews merely focused on the qualitative association between SES and adherence and did not deal with the influence of confounding factors. However, this study confirmed, quantified, and further explored the positive association between SES and adherence by performing meta-analysis and subgroup analysis and comprehensively interpreting the results of both the unadjusted association and the adjusted one.

A few limitations should be considered when interpreting the results. First, considerable heterogeneity existed and although we performed subgroup analysis and sensitivity analysis, it remained. The probable explanation of the heterogeneity was that in the absence of a widely recognized SES index, we observed different kinds of indexes and scores, which combined different indicators at different levels and used different weights to indicate SES. However, most indexes and scores incorporate the 3 SES indicators of income, education, and employment. The different start ages and stop ages used by the different programs resulted in differences in the mean age of the population, leading to differences in sample characteristics. Second, we recognized that all the studies included were carried out in the high-income setting even though we did not make a restriction on the inclusion criteria and actively supplement the literature collection from the reference lists. It might be caused by a limited number of studies that quantitatively assessed the association between SES and adherence to organized screening programs, especially in low-income countries where organized screening was not widespread. Therefore, whether the results can be generalized to low- and middle-income countries remains unknown. Finally, different adjustments might cause residual confounders in the included studies. To minimize this effect, we took both unadjusted and adjusted effect sizes into account and were more confident to draw a conclusion when the results of them were consistent.

### Conclusions

To conclude, we found the high-SES population had higher adherence to FOBT-based organized CRC screening. Neighborhood SES or small-area SES was more competent than individual- and country-level SES to be used to assess the association between SES and adherence. Race and ethnicity were probably important confounding factors for the association. The good news was that the disparity of adherence between the high SES and the low SES narrowed with the development of interventions and the improvement of organized programs. Future research should focus on digging into the causation of the association and creating targeted interventions and strategies to improve the adherence of the low-SES population, which aims at overcoming the inequality in the chances of benefiting from organized screening programs.

## Appendix

Multimedia Appendix 1 Supplementary Tables and Figures.

## Abbreviations

AHRQ: Agency for Healthcare Research and Quality
CRC: colorectal cancer
FOBT: fecal occult blood test
NOS: Newcastle-Ottawa Quality Assessment Scale
OR: odds ratio
PRISMA: Preferred Reporting Items for Systematic Reviews and Meta-Analyses
SES: socioeconomic status

## Data Availability

The data sets generated during and/or analyzed during this study are available from the corresponding author on reasonable request.
