# Supplementary material for: Association Between Socioeconomic Status and Adherence to Fecal Occult Blood Tests in Colorectal Cancer Screening Programs: Systematic Review and Meta-Analysis of Observational Studies
Source: JMIR Public Health Surveill. 2023 Oct 31;9:e48150. doi: 10.2196/48150 (PMC10646673; doi:10.2196/48150)
Supplement: Multimedia Appendix 1 [file publichealth_v9i1e48150_app1.docx]

Catalogue

**Supplementary Materials** 2

Supplementary Tables 2

Table S1. Search strategy. 2

Table S2. Supplementary overview of the studies included in the meta-analysis. 3

Table S3. Quality assessment of the articles according to the NewCastle Ottawa Scale for cohort studies. 4

Table S4. Agency for Healthcare Research and Quality (AHRQ) checklist for assessing the quality of cross-sectional studies. 4

Supplementary Figures 5

Figure S1. Sensitivity analysis of unadjusted effect sizes. 5

Figure S2. Sensitivity analysis of adjusted effect sizes. 6

Figure S3. Forest plot of adjusted effect sizes of subgroup analysis of SES level. 7

Figure S4. Forest plot of unadjusted effect sizes of subgroup analysis of year of print. 8

Figure S5. Forest plot of adjusted effect sizes of subgroup analysis of year of print. 9

# Supplementary Materials

# Supplementary Tables

# Table S1. Search strategy.

| **Databases** | **Search strategy** |
| --- | --- |
| PubMed | **#1** socioeconomic[TIAB] OR "socioeconomic status"[TIAB] OR SES[TIAB] OR income[TIAB] OR education[TIAB] OR occupation[TIAB] OR insurance[TIAB] OR "social status"[TIAB]  **#2** (colorect*[TIAB] OR bowel[TIAB]) AND (cancer*[TIAB]) AND (screen*[TI] OR "early diagnosis"[TI] OR "early detection"[TI]) AND (organized[TIAB] OR program*[TIAB])  **#3** uptake[TIAB] OR adherence[TIAB] OR nonadherence[TIAB] OR compliance[TIAB] OR noncompliance[TIAB] OR participation[TIAB] OR participating[TIAB] OR nonparticipation[TIAB] OR attendance[TIAB] OR nonattendance[TIAB] OR engagement[TIAB] OR determin*[TIAB] OR factor*[TIAB] OR associat*[TIAB]  **#1** AND **#2** AND **#3** |
| Embase | **#1** socioeconomic:ti,ab OR 'socioeconomic status':ti,ab OR ses:ti,ab OR income:ti,ab OR education:ti,ab OR occupation:ti,ab OR insurance:ti,ab OR 'social status':ti,ab  **#2** (colorect*:ti,ab OR bowel:ti,ab) AND cancer*:ti,ab AND (screen*:ti OR 'early diagnosis':ti OR 'early detection':ti) AND ('organized':ti,ab OR program*:ti,ab)  **#3** uptake:ti,ab OR adherence:ti,ab OR nonadherence:ti,ab OR compliance:ti,ab OR noncompliance:ti,ab OR participation:ti,ab OR participating:ti,ab OR nonparticipation:ti,ab OR attendance:ti,ab OR nonattendance:ti,ab OR engagement:ti,ab OR determin*:ti,ab OR factor*:ti,ab OR associat*:ti,ab  **#1** AND **#2** AND **#3** |
| Web of Science | **#1** TI=(socioeconomic OR "socioeconomic status" OR SES OR income OR education OR occupation OR insurance OR "social status") OR AB=(socioeconomic OR "socioeconomic status" OR SES OR income OR education OR occupation OR insurance OR "social status")  **#2** TI=(colorect* OR bowel) OR AB=(colorect* OR bowel)  **#3** TI=cancer* OR AB=cancer*  **#4** TI=(organized OR program*) OR AB=(organized OR program*)  **#5** TI=(screen* OR "early diagnosis" OR "early detection")  **#6** TI=(uptake OR adherence OR nonadherence OR compliance OR noncompliance OR participation OR participating OR nonparticipation OR attendance OR nonattendance OR engagement OR determin* OR factor* OR associat*) OR AB=(uptake OR adherence OR nonadherence OR compliance OR noncompliance OR participation OR participating OR nonparticipation OR attendance OR nonattendance OR engagement OR determin* OR factor* OR associat*)  **#1** AND **#2** AND **#3** AND **#4** AND **#5** AND **#6**  Indexes=SCI-EXPANDED, SSCI, A&HCI, CPCI-S, CPCI-SSH, ESCI Timespan=1970-2023 |

# Table S2. Supplementary overview of the studies included in the meta-analysis.

| **First author, year** | **Age range** | **Sample size** | **Adherence rate (%)** | **Study design** | **Adjusted effect size** | **Available estimates** | **Unadjusted OR (95%CI)** | **Adjusted OR (95%CI)** | **Covariates** | **Quality** |
| --- | --- | --- | --- | --- | --- | --- | --- | --- | --- | --- |
| Poncet 2013 [33] | 50-74 | 247,776 | 40.1 | Cohort | OR | Both | 1.32 (1.28, 1.35) | 1.33 (1.26, 1.41) | Age, sex, health insurance plan | High |
| Pornet 2010 [34] | 50-74 | 8691 | 34.7 | Cohort | OR | Both | 1.36 (1.18, 1.56) | 1.47 (1.27, 1.70) | Age, sex, insurance coverage | High |
| Solís-Ibinagagoitia 2020 [35] | 50-69 | 515,388 | 71.9 | Cross-sectional | OR | Adjusted | — | 1.15 (1.12, 1.17) | Age, sex, comorbidity, tobacco, diabetes, arterial hypertension, primary care visits | High |
| Steele 2010 [36] | 50-69 | 304,245 | 55.0 | Cohort | — | Unadjusted | 2.34 (2.27, 2.40) | — | — | High |
| Szczepura 2008 [37] | 50-69 | 207,417 | 51.2 | Cohort | OR | Both | 2.44 (2.36, 2.51) | 2.27 (2.20, 2.35) | Age, sex, ethnicity | High |
| van der Meulen 2022 [38] | 61-76 | 1,866,060 | 72.9 | Cohort | OR | Both | 1.40 (1.38, 1.41) | 1.37 (1.35, 1.39) | Age, sex | High |
| van der Vlugt 2017 [39] | 50-74 | 17312 | 72.0 | Cohort | — | Unadjusted | 2.13 (1.88, 2.42) | — | — | High |
| Ward 2011 [40] | 55-65 | 74,782 | 46.1 | Cohort | OR | Both | 1.39 (1.32, 1.45) | 1.44 (1.39, 1.53) | Sex, geographical accessibility | High |
| Weller 2007 [41] | 50-69 | 127,746 | 52.1 | Cohort | OR | Both | 2.66 (2.56, 2.77) | 2.44 (2.32, 2.56) | Age, sex, ethnicity | High |
| Buron 2017 [42] | 50-69 | 172962 | 44.7 | Cohort | OR | Both | 1.22 (1.19, 1.26) | 1.20 (1.16, 1.23) | Age, sex | High |

Age range: Age range of individuals invited to participate in screening.

OR, odds ratio.

# Table S3. Quality assessment of the articles according to the NewCastle Ottawa Scale for cohort studies.

| Study | Selection | | | | Comparability | Outcome | | | Overall | Quality |
| --- | --- | --- | --- | --- | --- | --- | --- | --- | --- | --- |
| Poncet 2013^1^ | 1 | 1 | 1 | 1 | 2 | 1 | 1 | 1 | 9 | High |
| Pornet 2010^2^ | 1 | 1 | 1 | 1 | 2 | 1 | 1 | 1 | 9 | High |
| Steele 2010^4^ | 1 | 1 | 1 | 1 | 0 | 1 | 1 | 1 | 7 | High |
| Szczepura 2008^5^ | 1 | 1 | 1 | 1 | 1 | 1 | 1 | 1 | 8 | High |
| van der Meulen 2022^6^ | 1 | 1 | 1 | 1 | 1 | 1 | 1 | 1 | 8 | High |
| van der Vlugt 2017^7^ | 1 | 1 | 1 | 1 | 0 | 1 | 1 | 1 | 7 | High |
| Ward 2011^8^ | 1 | 1 | 1 | 1 | 1 | 1 | 1 | 1 | 8 | High |
| Weller 2007^9^ | 1 | 1 | 1 | 1 | 1 | 1 | 1 | 1 | 8 | High |
| Buron 2017^10^ | 1 | 1 | 1 | 1 | 1 | 1 | 1 | 1 | 8 | High |

The NewCastle Ottawa Scale for cohort studies includes selection, comparability and outcome with 8 items. Selection: 1) Representativeness of the exposed cohort; 2) Selection of the non exposed cohort; 3) Ascertainment of exposure; 4) Demonstration that outcome of interest was not present at start of study. Comparability: 1) Comparability of cohorts on the basis of the design or analysis: whether or not the study controls for the most important factor and any additional factor. Outcome: 1) Assessment of outcome; 2) Was follow-up long enough for outcomes to occur; 3) Adequacy of follow up of cohorts.

# Table S4. Agency for Healthcare Research and Quality (AHRQ) checklist for assessing the quality of cross-sectional studies.

| Study | Score | | | | | | | | | | | | Quality |
| --- | --- | --- | --- | --- | --- | --- | --- | --- | --- | --- | --- | --- | --- |
|  | Item 1 | Item 2 | Item 3 | Item 4 | Item 5 | Item 6 | Item 7 | Item 8 | Item 9 | Item 10 | Item 11 | Total |  |
| Solís-Ibinagagoitia 2020^3^ | 1 | 1 | 1 | 1 | 0 | 0 | 1 | 1 | 0 | 0 | 0 | 6 | High |

The Agency for Healthcare Research and Quality (AHRQ) methodology checklist includes 11 items: 1) Define the source of information (survey, record review); 2) List inclusion and exclusion criteria for exposed and unexposed subjects (cases and controls) or refer to previous publications; 3) Indicate time period used for identifying patients; 4) Indicate whether or not subjects were consecutive if not population-based; 5) Indicate if evaluators of subjective components of study were masked to other aspects of the status of the participants; 6) Describe any assessments undertaken for quality assurance purposes (e.g., test/retest of primary outcome measurements); 6) Describe any assessments undertaken for quality assurance purposes (e.g., test/retest of primary outcome measurements); 7) Explain any patient exclusions from analysis; 8) Describe how confounding was assessed and/or controlled; 9) If applicable, explain how missing data were handled in the analysis; 10) Summarize patient response rates and completeness of data collection; 11) Clarify what follow-up, if any, was expected and the percentage of patients for which incomplete data or follow-up was obtained. An item was scored ‘0’ with the risk of bias classified as ‘high’ or ‘unclear’ and scored ‘1’ if it was answered ‘low’.

# Supplementary Figures

# Figure S1. Sensitivity analysis of unadjusted effect sizes.


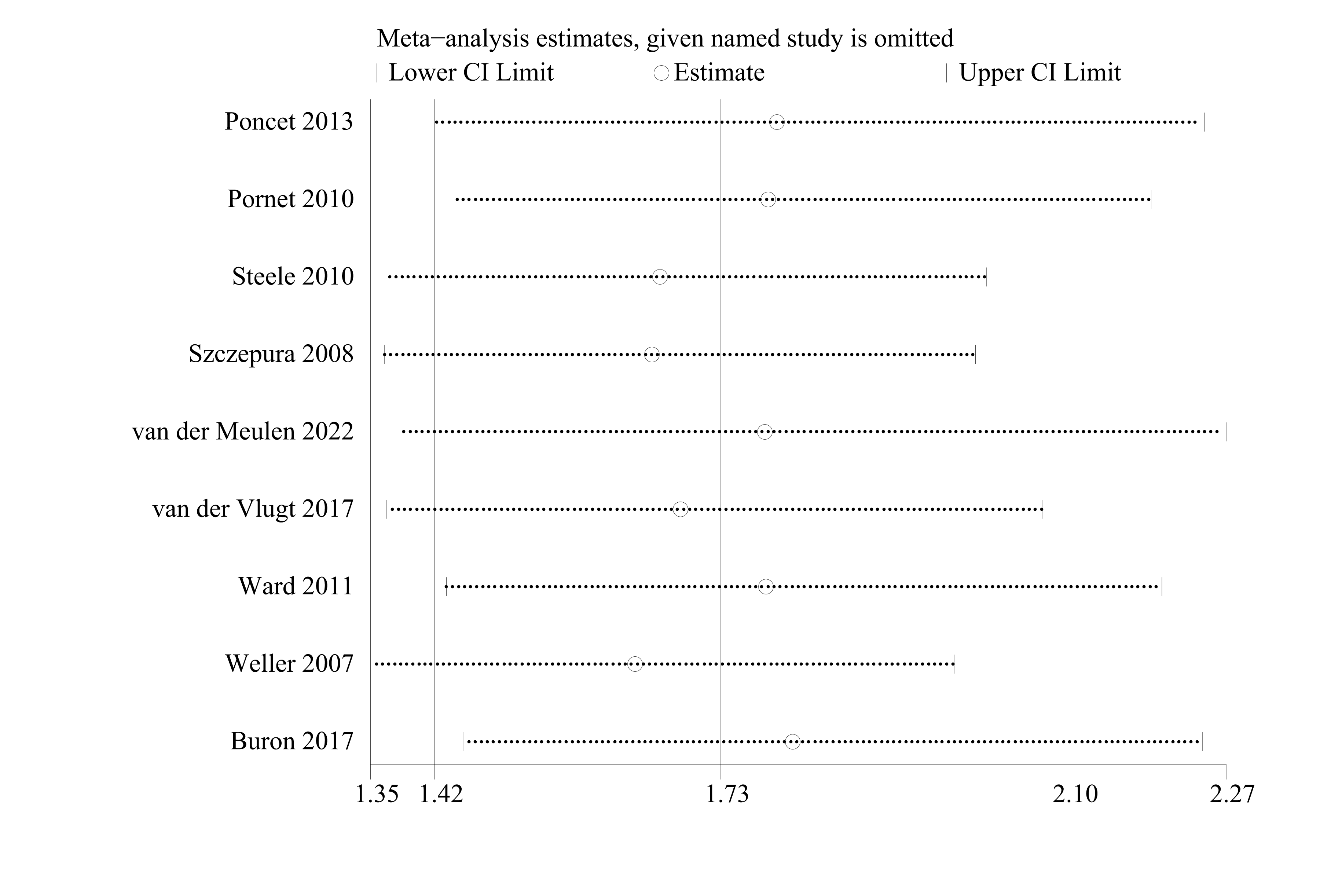


# Figure S2. Sensitivity analysis of adjusted effect sizes.


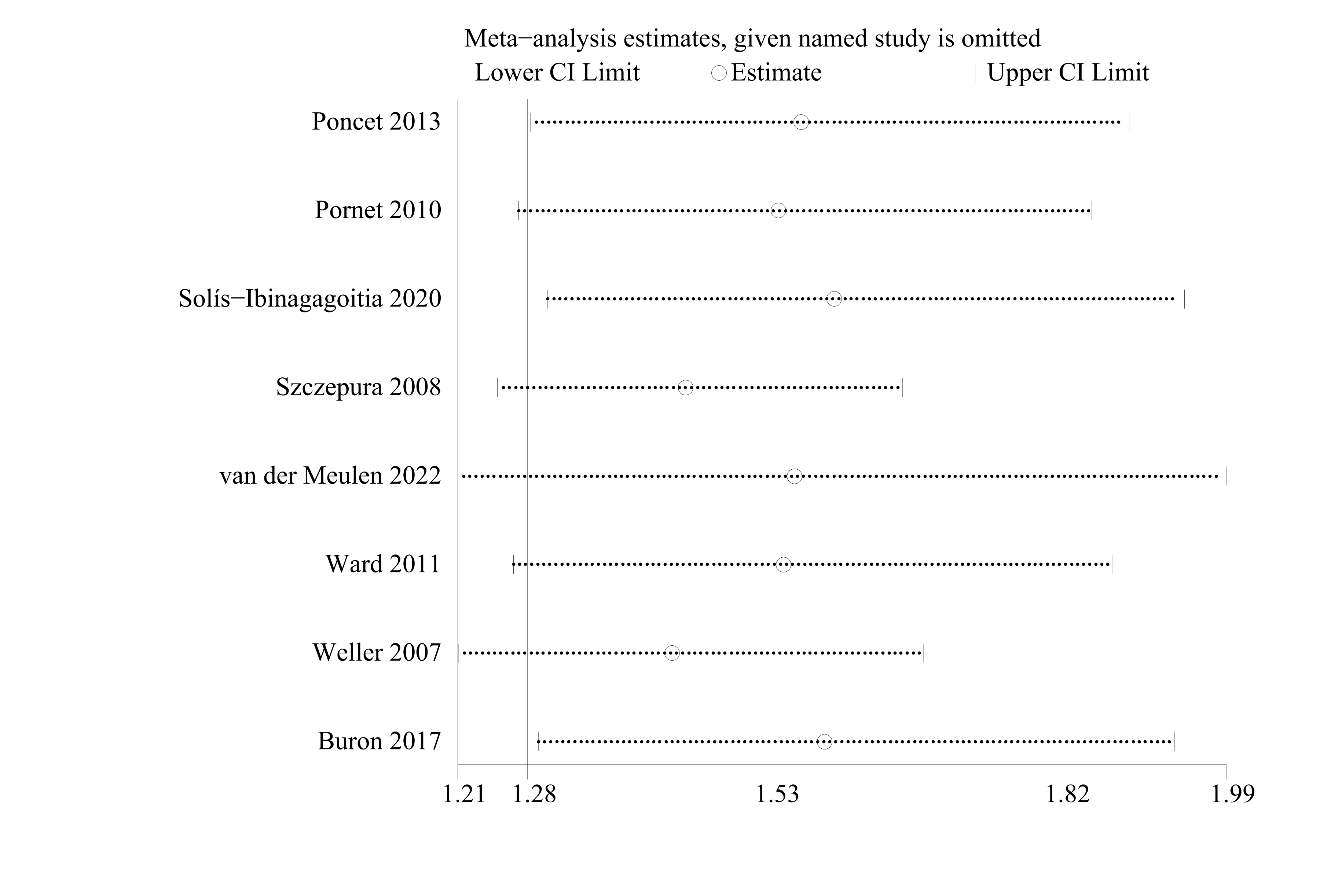


# Figure S3. Forest plot of adjusted effect sizes of subgroup analysis of SES level. OR: odds ratio.


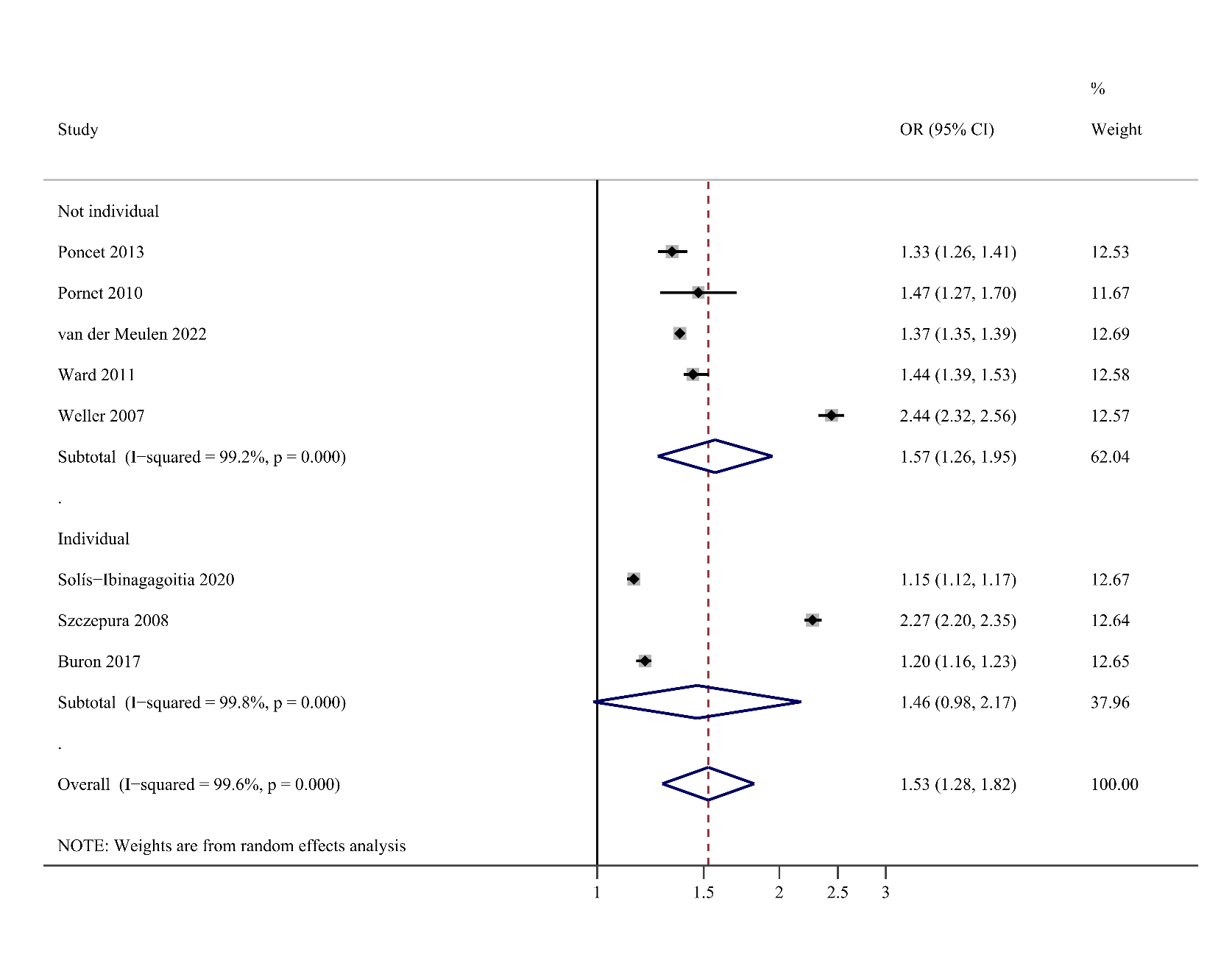


# Figure S4. Forest plot of unadjusted effect sizes of subgroup analysis of year of print. OR: odds ratio.


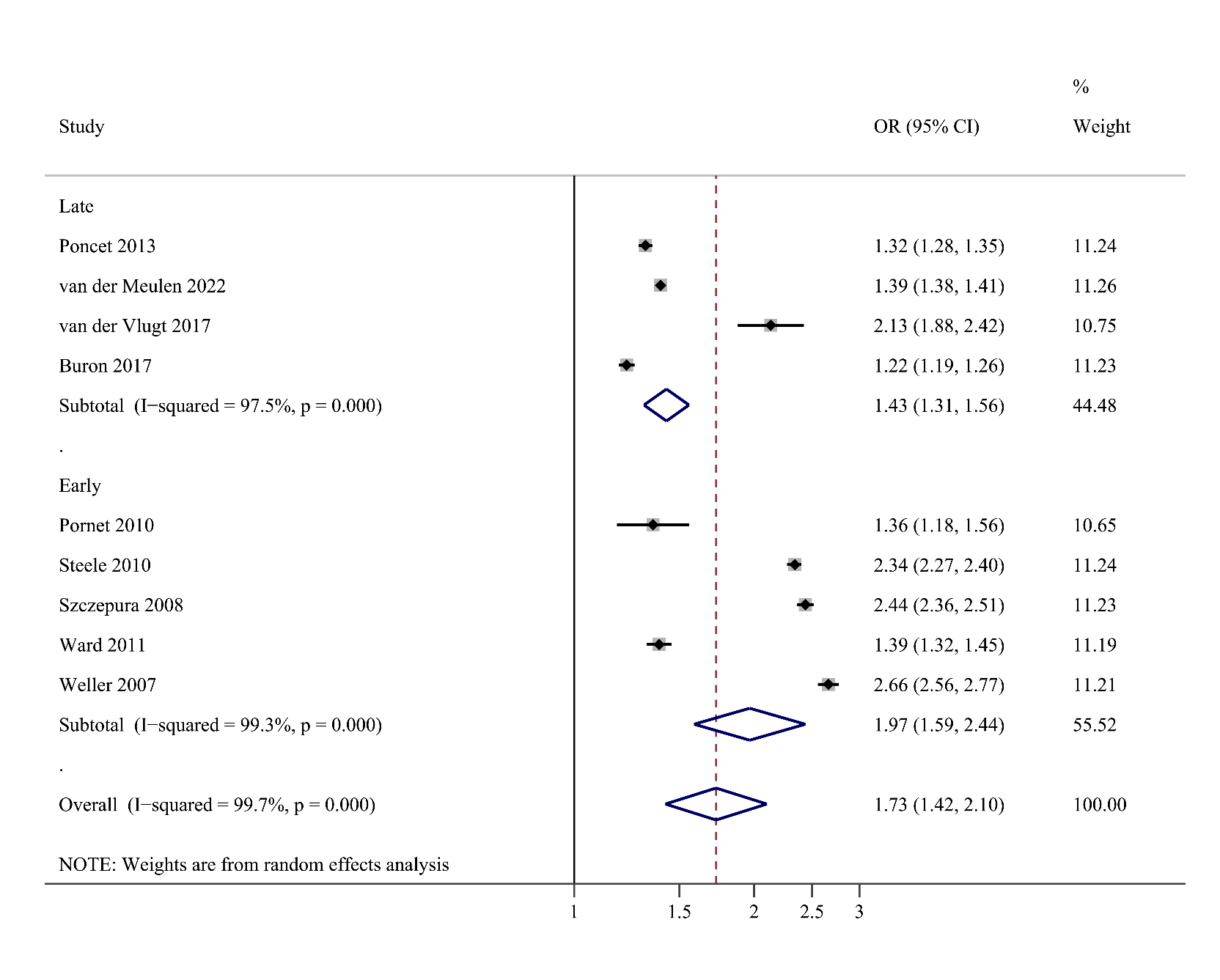


# Figure S5. Forest plot of adjusted effect sizes of subgroup analysis of year of print. OR: odds ratio.


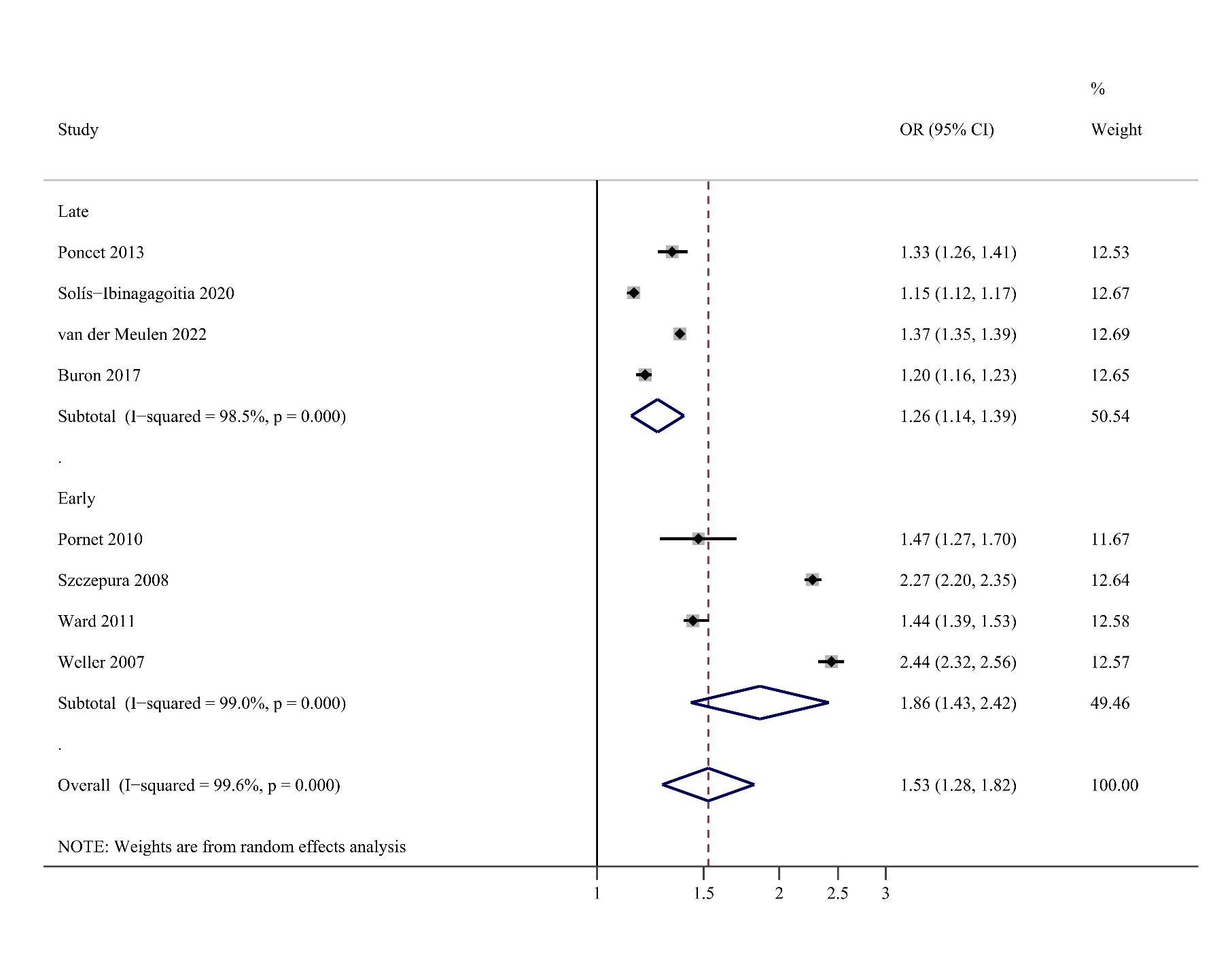


**References**

1. Poncet F, Delafosse P, Seigneurin A, Exbrayat C, Colonna M. Determinants of participation in organized colorectal cancer screening in Isère (France). *Clinics and research in hepatology and gastroenterology* 2013; **37**(2): 193-9.

2. Pornet C, Dejardin O, Morlais F, Bouvier V, Launoy G. Socioeconomic determinants for compliance to colorectal cancer screening. A multilevel analysis. *Journal of epidemiology and community health* 2010; **64**(4): 318-24.

3. Solís-Ibinagagoitia M, Unanue-Arza S, Díaz-Seoane M, et al. Factors Related to Non-participation in the Basque Country Colorectal Cancer Screening Programme. *Frontiers in public health* 2020; **8**: 604385.

4. Steele RJ, Kostourou I, McClements P, et al. Effect of gender, age and deprivation on key performance indicators in a FOBT-based colorectal screening programme. *Journal of medical screening* 2010; **17**(2): 68-74.

5. Szczepura A, Price C, Gumber A. Breast and bowel cancer screening uptake patterns over 15 years for UK south Asian ethnic minority populations, corrected for differences in socio-demographic characteristics. *BMC public health* 2008; **8**: 346.

6. van der Meulen MP, Toes-Zoutendijk E, Spaander MCW, et al. Socioeconomic differences in participation and diagnostic yield within the Dutch national colorectal cancer screening programme with faecal immunochemical testing. *PloS one* 2022; **17**(2): e0264067.

7. van der Vlugt M, Grobbee EJ, Bossuyt PM, et al. Adherence to colorectal cancer screening: four rounds of faecal immunochemical test-based screening. *British journal of cancer* 2017; **116**(1): 44-9.

8. Ward PR, Javanparast S, Matt MA, et al. Equity of colorectal cancer screening: cross-sectional analysis of National Bowel Cancer Screening Program data for South Australia. *Australian and New Zealand journal of public health* 2011; **35**(1): 61-5.

9. Weller D, Coleman D, Robertson R, et al. The UK colorectal cancer screening pilot: results of the second round of screening in England. *British journal of cancer* 2007; **97**(12): 1601-5.

10. Buron A, Auge JM, Sala M, et al. Association between socioeconomic deprivation and colorectal cancer screening outcomes: Low uptake rates among the most and least deprived people. *PloS one* 2017; **12**(6): e0179864.
